# The inguinal region revisited: the surgical point of view: An anatomical–surgical mapping and sonographic approach regarding postoperative chronic groin pain following open hernia repair

**DOI:** 10.1007/s10029-019-02070-z

**Published:** 2019-11-27

**Authors:** M. Konschake, M. Zwierzina, B. Moriggl, R. Függer, F. Mayer, W. Brunner, T. Schmid, D. C. Chen, R. Fortelny

**Affiliations:** 1 Department of Anatomy, Histology and Embryology, Division of Clinical and Functional Anatomy, Medical University of Innsbruck, Müllerstr. 59, 6020 Innsbruck, Austria; 2 Department of Plastic, Reconstructive and Aesthetic Surgery, Center of Operative Medicine, Medical University of Innsbruck, Innsbruck, Austria; 3 Department of Surgery, Elisabethinen Hospital, Linz, Austria; 4 Department of Surgery, Paracelsus Medical University, Salzburg, Austria; 5 Department of Surgery, Kantonspital St. Gallen, St. Gallen, Switzerland; 6 Department for Visceral-, Transplantation- and Thoracic Surgery, Center of Operative Medicine, Medical University of Innsbruck, Innsbruck, Austria; 7 Department of Surgery, David Geffen School of Medicine at UCLA, Los Angeles, CA USA; 8 Lichtenstein Amid Hernia Clinic, Santa Monica, CA USA; 9 Department of General-, Visceral- and Oncological Surgery, Wilhelminenspital, Vienna, Austria

**Keywords:** Chronic groin pain, Open hernia repair, Inguinal nerves, Inguinodynia, Ultrasound

## Abstract

**Purpose:**

Inguinodynia or chronic post-herniorrhaphy pain, defined as pain lasting longer than 3 months after open inguinal hernia repair, has become the most important complication after inguinal surgery and therefore compromises the patient´s quality of life. A major reason for inguinodynia might be the lack of neuroanatomical knowledge and suboptimal “management” of the nerves during surgery.

**Methods:**

We present a detailed neuroanatomic mapping of the inguinal region by dissection including the most important surgical landmarks with all nerves confirmed by immunohistochemistry, ultrasound guided visualization of the iliohypogastric, ilio-inguinal, and genital branch of the genitofemoral nerve, and a practical (preoperative) algorithm for clinical management.

**Results:**

Surgically and ultrasonographically relevant structures (“landmarks”) in open hernia repair are the anterior–superior iliac spine, pubic tubercle, Camper´s fascia (superficial layer of the superficial abdominal fascia), External oblique aponeurosis, Internal oblique muscle, Transversus abdominis muscle, superficial inguinal ring, external spermatic fascia, cremasteric fascia with cremaster muscle fibers, internal spermatic fascia, cremasteric vein (=external spermatic vein = “blue line”), ductus deferens, pampiniform plexus, inguinal ligament and the inferior epigastric vessels.

**Conclusion:**

A detailed understanding of inguinal anatomy is an indispensable basic requirement for all surgeons to perform inguinal ultrasonography as well as open inguinal hernia repair, avoiding complications, especially postoperative inguinodynia.

## Introduction

The International Association for the Study of Pain (IASP) defines chronic pain as pain arising at or beyond 3 months after injury [1, 2]. Regardless, this general definition can be applied to pain caused by surgical interventions. Therefore, inguinodynia or the post herniorrhaphy pain syndrome (PHPS) is defined as pain or discomfort lasting longer than three months after inguinal hernia repair. Pain may arise as a direct consequence of a nerve lesion or a disease affecting the somatosensory system in patients with no prior history of groin pain before their original hernia surgery. If there is a history of groin pain, the post-operative condition must be clearly differentiated to qualify as PHPS [1].

PHPS has an incidence of 0–69% [3–10] and can be differentiated further into nociceptive and neuropathic pain. Neuropathic pain defined as pain caused by direct nerve injury and is characterized by sensory dysfunction in the surgical area. On the other hand, nociceptive pain is caused by tissue injury or inflammatory reaction [1, 11, 12]. Nevertheless, a clear differentiation between the two subgroups of PHPS after herniorrhaphy is difficult, owing to the unclear definition and terminology of neuropathic pain in the literature [1, 4–7, 11–15]. There only seems to be agreement in the use of validated questionnaires and score systems to assess pain at rest and during well-defined daily activities [3]. Furthermore, O´Dwyer and Kehlet et al. note that nerve damage during surgery is a prerequisite for the development of a PHPS [16, 17]. Table 1, according to the study of Bay-Nielsen et al. [3], shows data of 2612 patients with a good overview of the incidence of chronic postherniorrhaphy pain after three different open hernia repair techniques (Lichtenstein, Shouldice, Marcy).

**Table 1:** The incidence of pain, discomfort different from pain, infection and a new bulge in the operated groin in relation to the type of repair (according to Bay-Nielsen et al. [3])

	Lichtenstein (*n* = 1.250)	Shouldice (*n* = 630)	Marcy (*n* = 732)	Total (*n* = 2.612)
Total Pain within previous month	316 (25.3%)	119 (18.9%)	162 (22.1%)	597 (22.9%)
Discomfort different from pain within previous month	245 (19.6%)	119 (18.9%)	122 (16.7%)	486 (18.6%)
Wound infection	37 (3%)	21 (3.3%)	20 (2.7%)	78 (3%)
New bulge in groin	80 (6.4%)	43 (6.8%)	59 (8.1%)	182 (7%)

Only a few studies exist in the literature highlighting the topography of all three inguinal nerves [18–20]. Most of the studies only investigate one or two inguinal nerves [21–24] or concentrate on ultrasound-guided nerve pain blocks before and/or after surgery [22, 23, 25].

To the best of our knowledge, there is no work investigating the exact neuroanatomy of all three inguinal nerves and the spermatic cord sheaths throughout all surgical layers including an algorithm of an ultrasonographic approach to visualize the iliohypogastric (IHN), ilio-inguinal (I-IN) and genital branch of the genitofemoral nerve (GBGFN) for routine daily practice.

Therefore, the aim of our study was to illustrate a comprehensible mapping of the neuroanatomy of the inguinal region and the spermatic cord sheaths by anatomical cadaveric dissection and intraoperative observation, along with ultrasonographic visualization of the IHN, I-IN and GBGFN.

## Materials and methods

### Macroscopic dissection

To demonstrate the surgical layers, their relationship to the anatomical landmarks and the topography of all three inguinal nerves during open inguinal hernia surgery, we dissected two male specimens embalmed with a formaldehyde–phenol solution [26, 27].

The bodies were donated to the Division of Clinical and Functional Anatomy of the Medical University of Innsbruck. All donors had given their written informed consent for their use for scientific and educational purposes prior to death [28, 29]. According to Austrian National Law, scientific institutions (in general Institutes, Departments or Divisions of Medical Universities) are entitled to receive the body after death mainly by means of a specific legacy, which is a special form of last will and testament. No bequests are accepted without the donor having registered their legacy and been given appropriate information, before they make a decision based upon written informed consent (policy of ethics) [30]; therefore, an ethics committee approval is not necessary.

Additionally, we performed two open tension-free hernioplasties (Lichtenstein technique) [31]. Surgery was performed on two male patients with unilateral direct inguinal hernias admitted to the Department for Visceral, Transplantation and Thoracic Surgery of the Medical University of Innsbruck. Written informed consent was obtained before surgery. Hernia repairs were performed by an experienced surgeon. Both patients had no prior history of groin pain or numbness.

The anatomical and clinical investigations were photo-documented to develop a clear and understandable anatomical mapping of the inguinal region.

### Immunohistochemistry of the three inguinal nerves

To confirm that inguinal nerves identified macroscopically were in fact nerve tissue, we additionally produced microscopic sections of the presumed nerves. Specimens were excised and then promptly fixed in ice-cold 4% paraformaldehyde (PFA) in phosphate-buffered saline (PBS, 0.1 M) at a pH of 7.4 and left overnight. Subsequently, the specimens were rinsed in PBS and were then prepared by dehydrating and later embedding in paraffin using a histological infiltration processor (Miles Scientific Inc., Naperville, IL, USA). Sequential sections of 4 μm thickness were made on a HM 355S microtome (Microm, Walldorf, Germany) and affixed on SuperFrost® Plus slides (Menzel, Braunschweig, Germany). The affixed specimens were dried overnight at room temperature. Afterwards, the section-containing slides were incubated at 60 °C for 2 h to adhere the sectioned specimens firmly onto them.

Immunohistochemistry was rendered with a Ventana Roche® Discovery XT Immunostainer (Mannheim, Germany), using a DAB-MAP discovery research standard procedure.

The mounted sections were incubated with the appropriate primary antibody (S100-antibody) at 37 °C for 1 h. Following this the specimens were incubated with Discovery Universal Secondary Antibody, Ventana 760–4250 at room temperature for 30 min. Antibody detection was attained with the DAB-MAP Detection Kit (Ventana 760–124) using a combinatorial approach involving the diaminobenzidine development method with copper enhancement followed by light counter staining with haematoxylin (Ventana 760–2021) for 4 min. The stained sections were then manually dehydrated using an upgraded alcohol series, clarified with xylene and then mounted permanently with Entellan® (Merck, Darmstadt, Germany).

The entire immunohistochemical staining reaction was benchmarked against appositive controls (e.g., small intestine, brain, and pancreas). Auxiliary negative controls were acquired by alternating the primary antibodies with reaction buffer or substituting them with isotype matching immunoglobulins.

### Ultrasound guided visualization of the three inguinal nerves

For ultrasound visualization, we used an 18–6 MHz linear transducer (LA435; system MyLab25 by Esaote, Genoa, Italy), utilizing the highest frequency. Every inguinal nerve could be scanned and displayed at a so-called “point of optimal visibility” (“POV”) [32].

### Iliohypogastric and Ilio-inguinal nerve (IHN, I-IN) (Fig. 1a, b)

**Fig. 1:**
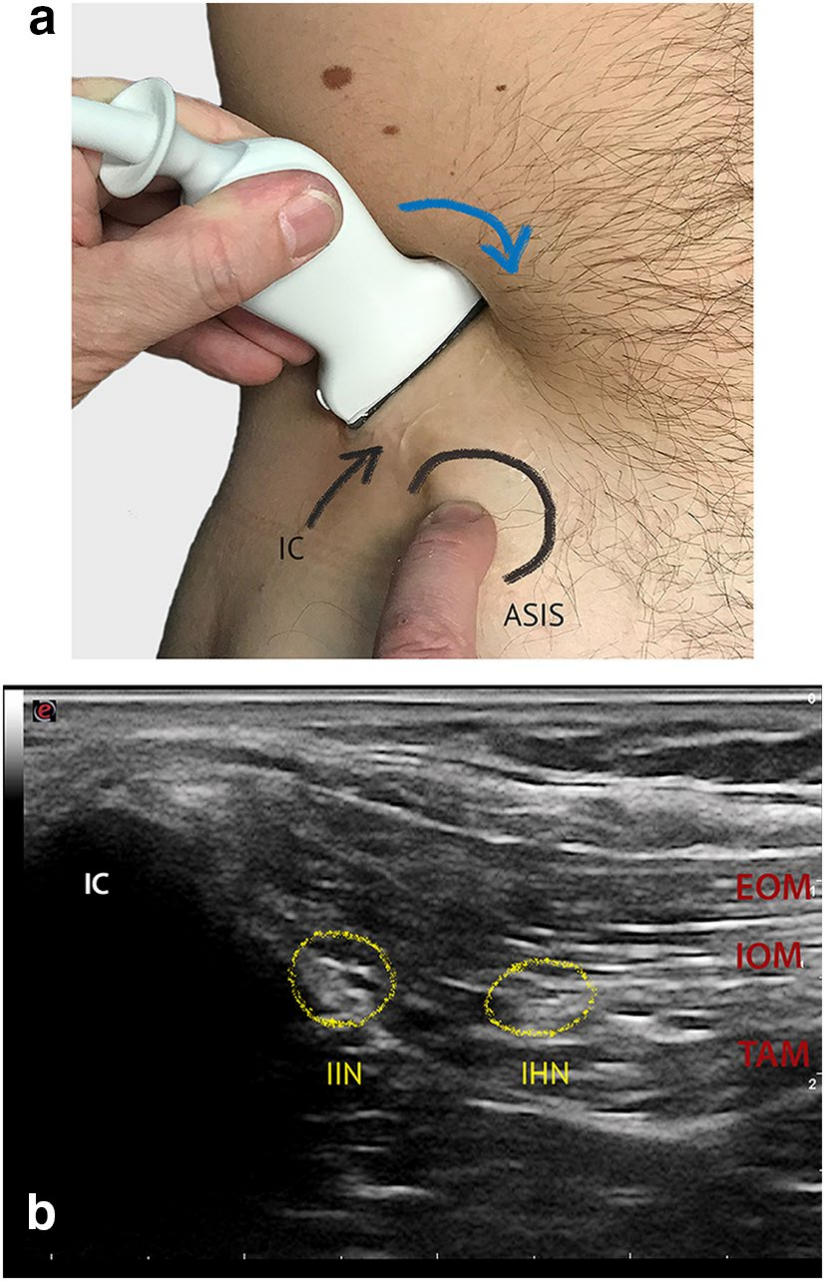
Probe placement for high-resolution ultrasonographic visualization of IHN and I-IN (ESAOTE, Italy, 13 MHz, linear array probe). a The arrow indicates the probe movement. The ASIS (black circle) and IC as landmarks. b Ultrasonographic image of I-IN and IHN obtained by the probe position. I-IN and IHN (yellow circles) lying in between the Internal oblique (IOM) and Transversal abdominal muscle (TAM). *IC* iliac crest

The volunteer was positioned supine. The right abdominal wall was scanned about 5 cm cranial and lateral to the anterior superior iliac spine (Fig. 1a). This region was chosen because the IHN and the I-IN have penetrated the Transverse abdominal muscle at this location with a probability of 95% and 90%, respectively [33]. The IHN and the I-IN are found there in 90% of cases between the Transverse abdominal and Internal oblique muscle [33] (Fig. 1b). At this point, all three muscle layers forming the lateral abdominal wall (External oblique, Internal oblique and Transverse abdominal muscles) could be illustrated (Fig. 1b). The transducer was positioned in a slightly oblique plane to be perpendicular to the course of the IHN and I-IN (Fig. 1a). The lateral caudal part of the transducer was brought into contact with the iliac crest. Both nerves appeared as oval hypoechoic areas with hyperechoic spots, encircled by a hyperechoic horizon, showing the typical ultrasonographic appearance of peripheral nerves [23, 34–36]. Therefore, the “point of optimal visibility” (“POV”) for displaying the IHN and I-IN is the layer between the Internal oblique and Transverse abdominis muscle.

### Genital branch of the genitofemoral nerve (GBGFN) (Fig. 2a, b)

**Fig. 2:**
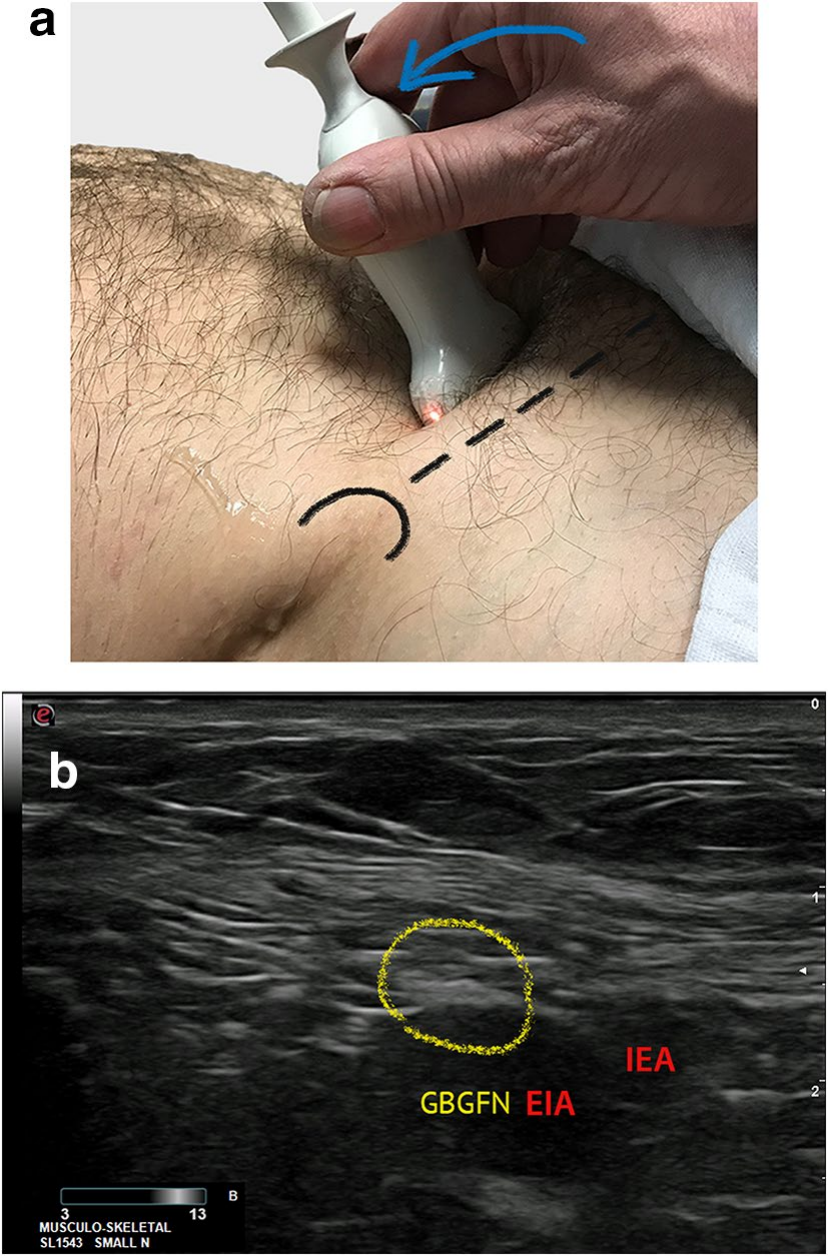
Probe placement for high-resolution ultrasonographic visualization of GBGFN (ESAOTE, Italy, 13 MHz, linear array probe). a The arrow indicates the probe movement. The ASIS (black semi-circle) and inguinal ligament as landmarks. b Ultrasonographic image of GBGFN obtained by the probe position. GBGFN (yellow circles). *EIA* external iliac artery, *GBGFN* genital branch of genital femoral nerve, *IEA* inferior epigastric artery

The volunteer was lying in supine position. The GBGFN was scanned about 2 cm cranial of the middle of the inguinal ligament, beginning the scanning at the anterior superior iliac spine laterally (Fig. 2a). The external iliac artery and the inferior epigastric artery can be displayed. The transducer was positioned in a slightly oblique plane to be perpendicular to the course of the GBGFN (Fig. 2a). Therefore the point of optimal visibility for the GBGFN is 2–3 cm cranial to the origin of the inferior epigastric artery, lying (regularly) superficially to the external iliac artery (Fig. 2a, b).

## Results

### Normal anatomy of the IHN, I-IN and GFN (Figs. 3, 12a, b)

**Fig. 3:**
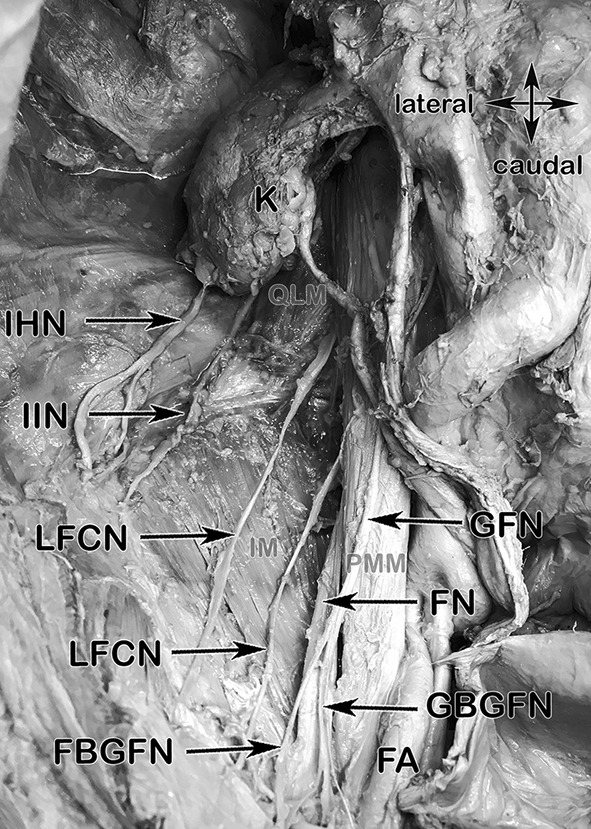
Anatomical specimen showing a topographic, retroperitoneal overview of the inguinal nerves, their courses and muscular landmarks. *K* kidney, *SCN* subcostal nerve, *IHN* iliohypogastric nerve, *I-IN* ilioinguinal nerve, *LFCNs* two branches of the lateral femoral cutaneous nerve, *FBGFN* femoral branch of genital femoral nerve, *GBGFN* genital branch of genital femoral nerve, *FA* femoral artery, *FN* femoral nerve, *GFN* genitofemoral nerve, *QLM* quadratus lumborum muscle (light brown), *IM* Iliacus muscle (light brown), *PMM* Psoas major muscle (light brown)

The IHN and I-IN, mixed motor and sensory nerves, derive from L1 and L1 nerve roots respectively. They take their course ventrally to the Quadratus lumborum muscle lying dorsally to the kidney. The IHN and I-IN penetrate the Transversus abdominis muscle in 61% at the dorsal segment of the iliac crest [33]. In 34.2% of the cases the IHN and the I-IN form a common trunk [37]. The furcation is possible on different topographic locations, dorsal of the kidney, ventral to the Quadratus lumborum muscle or directly at the level of the iliac crest at the penetration point of the Transversus abdominis muscle. After penetration, both nerves take their descending course ventro-medially in between the Internal oblique and Transverse abdominis muscle before piercing the Internal oblique muscle in the inguinal canal to lie deep to the External oblique aponeurosis. (Fig. 3).

The GFN derives from segment L1 and L2 and travels directly through the Psoas major muscle. It can penetrate this muscle as a common trunk or already divided into two branches, the genital branch and the femoral branch, and courses caudally, lying directly on the psoas major muscle (Fig. 3). The genital branch passes through the deep inguinal ring and descends within the spermatic cord supplying the Cremaster and Dartos muscle in males. In females it accompanies the round ligament and supplies the labia majora and mons pubis. The femoral branch passes deep to the inguinal ligament, travelling through the lacuna musculorum innervating the skin of the upper, anterior and medial side of the thigh.

### Gross anatomical findings: anatomical mapping

We dissected the cadaveric specimens and compared the findings to the two patients undergoing open inguinal hernia repairs to establish the anatomical mapping of all the important structures following the surgical steps:

#### Step 1: Anterior superior iliac spine (ASIS), pubic tubercle (PT) (Figs. 4, 12a, b)

**Fig. 4:**
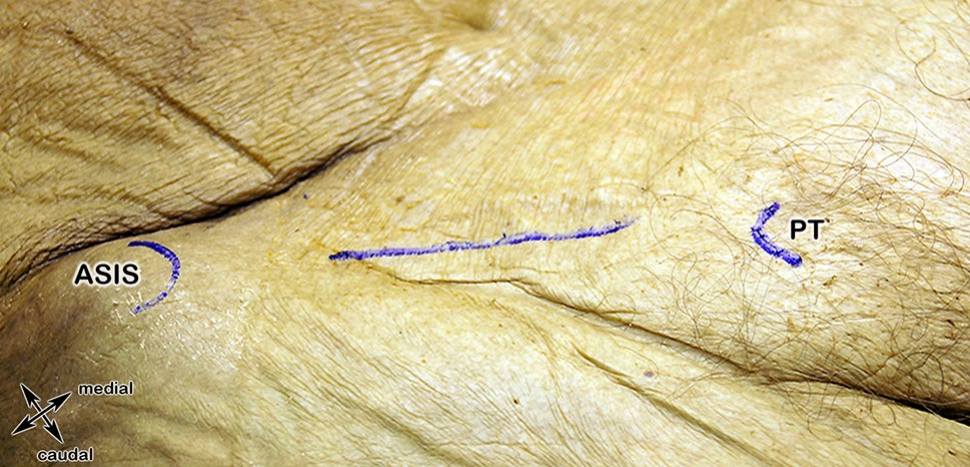
Anatomical specimen showing the first two landmarks of step 1 and the surgical cutting line 2 cm above the inguinal ligament. *ASIS* anterior superior iliac spine, *PT* pubic tubercle

At the beginning of the procedure, the cadaveric specimen was laid in supine position. The anterior superior iliac spine (ASIS), the pubic tubercle (PT) and the incision line, 2 cm cranially and parallel to the inguinal ligament, which can be regarded as the caudal part of the External oblique abdominal muscle, were marked with a surgical marker and skin was incised (Fig. 4). The pattern of the sensory IHN and I-IN branches piercing the External oblique aponeurosis should always be kept in mind (Fig. 12a, b).

#### Step 2: Camper´s fascia, Superficial inguinal ring (SIR) (Fig. 5)

**Fig. 5:**
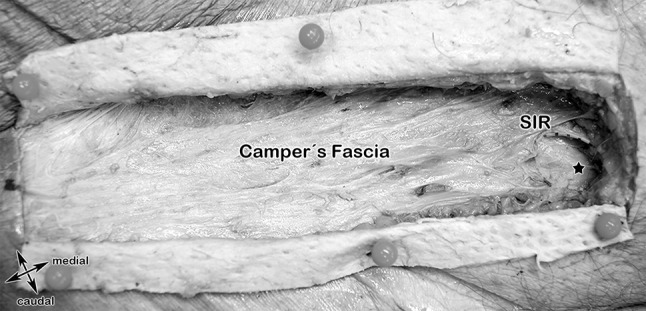
Anatomical specimen showing the step 2 landmarks. Camper's Fascia and the superficial inguinal ring (SIR), black star, spermatic cord

After dissecting the skin, the superficial fascia, also known as *Camper´s fascia* (defined as the superficial adipose tissue layer of the anterior abdominal wall, in contrast to *Scarpa’s* fascia, which is defined as the deep membranous layer of the superficial fascia of the abdomen) was identified. It was most prominent in the lower aspects of the abdominal wall below the level of the umbilicus. It contains a varying quantity of adipose tissue. Medial and inferior to the pubic tubercle, Camper's fascia in the male changes as it continues over the scrotum and forms dartos tunic. The superficial inguinal ring (SIR) could be then easily identified by palpation along the spermatic cord (Fig. 5).

#### Step 3: Superficial inguinal ring, IHN, I-IN (Figs. 6a, b, 7)

**Fig. 6:**
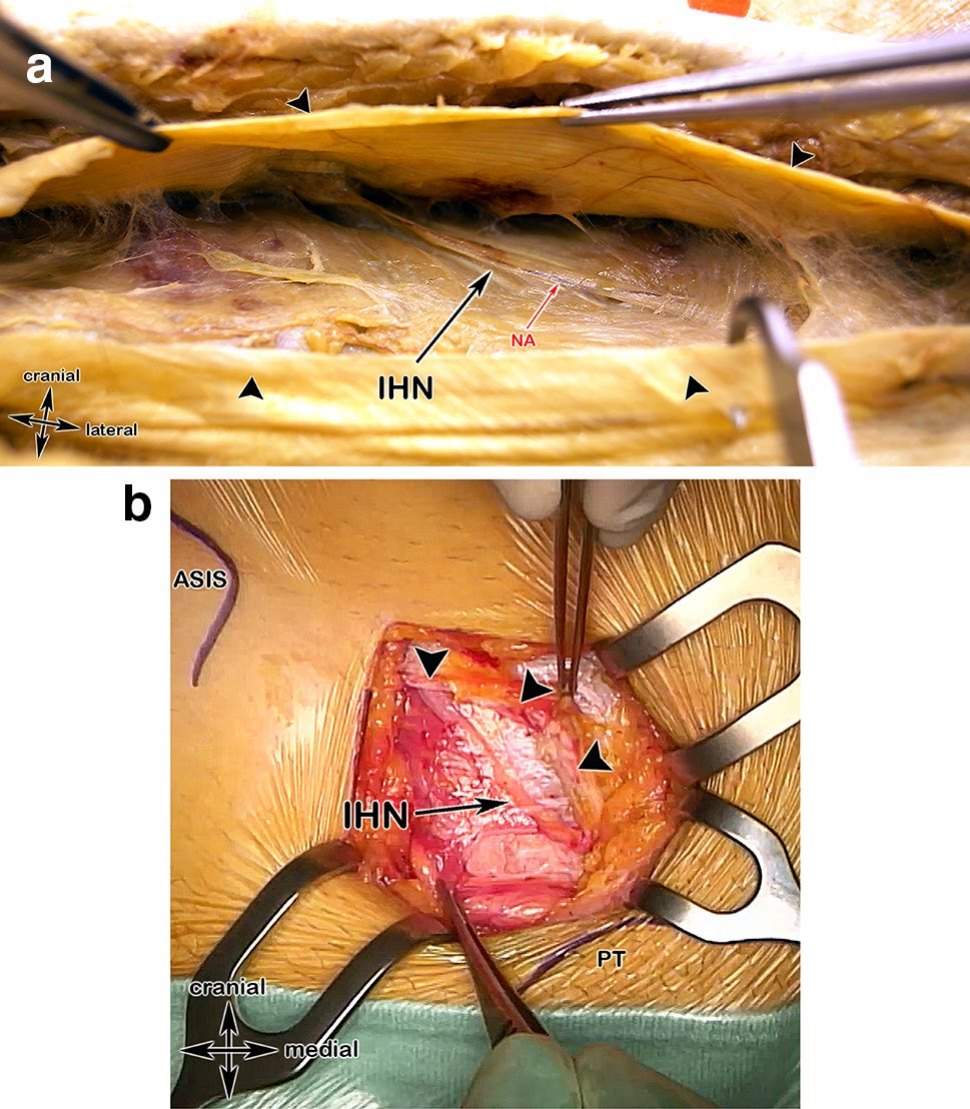
Anatomical specimen and a surgical case showing the step 3 landmarks. a After incision of the external oblique aponeurosis (black arrows), the iliohypogastric nerve, accompanied by its nutrient vessel and embedded in its connective tissue, became visible. *IHN* iliohypogastric nerve, *NA* nutrient artery. b Surgical case showing the anterior superior iliac spine (ASIS), the pubic tubercle (PT) and the iliohypogastric nerve after incision of the External oblique aponeurosis (black arrow heads)

**Fig. 7:**
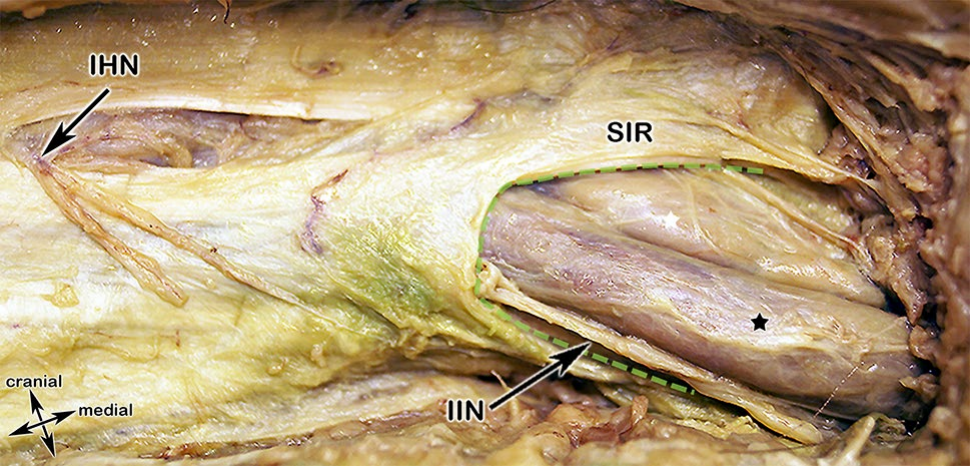
Anatomical specimen overview at the area of the superficial inguinal ring. After removing the Camper´s Fascia (= superficial layer of the superficial abdominal fascia), the medial branches of the iliohypogastric nerve piercing the aponeurosis of the External oblique muscle (in this specimen, at this topographic position, as an anatomical variant). The ilioinguinal nerve passes, together with the spermatic cord, through the superficial inguinal ring. IHN, iliohypogastric nerve; I-IN, ilio-inguinal nerve; SIR, superficial inguinal ring (green dashed line); black star, spermatic cord

In the next step, after identification of the superficial inguinal ring, the External oblique aponeurosis was incised. The incision was made parallel to the incision of the skin. The IHN could be therefore displayed easily and is embedded in the connective adipose tissue beneath the External oblique aponeurosis. (Fig. 6a, b) The I-IN could be displayed laterally at the superficial inguinal ring, laying lateral and superficial to the spermatic cord running in a descending course (Fig. 7).

#### Step 4: Exposure of the spermatic cord layers, IHN and I-IN, genital branch of the genitofemoral nerve (GBGFN), “Blue Line” (Fig. 8a–c)

**Fig. 8:**
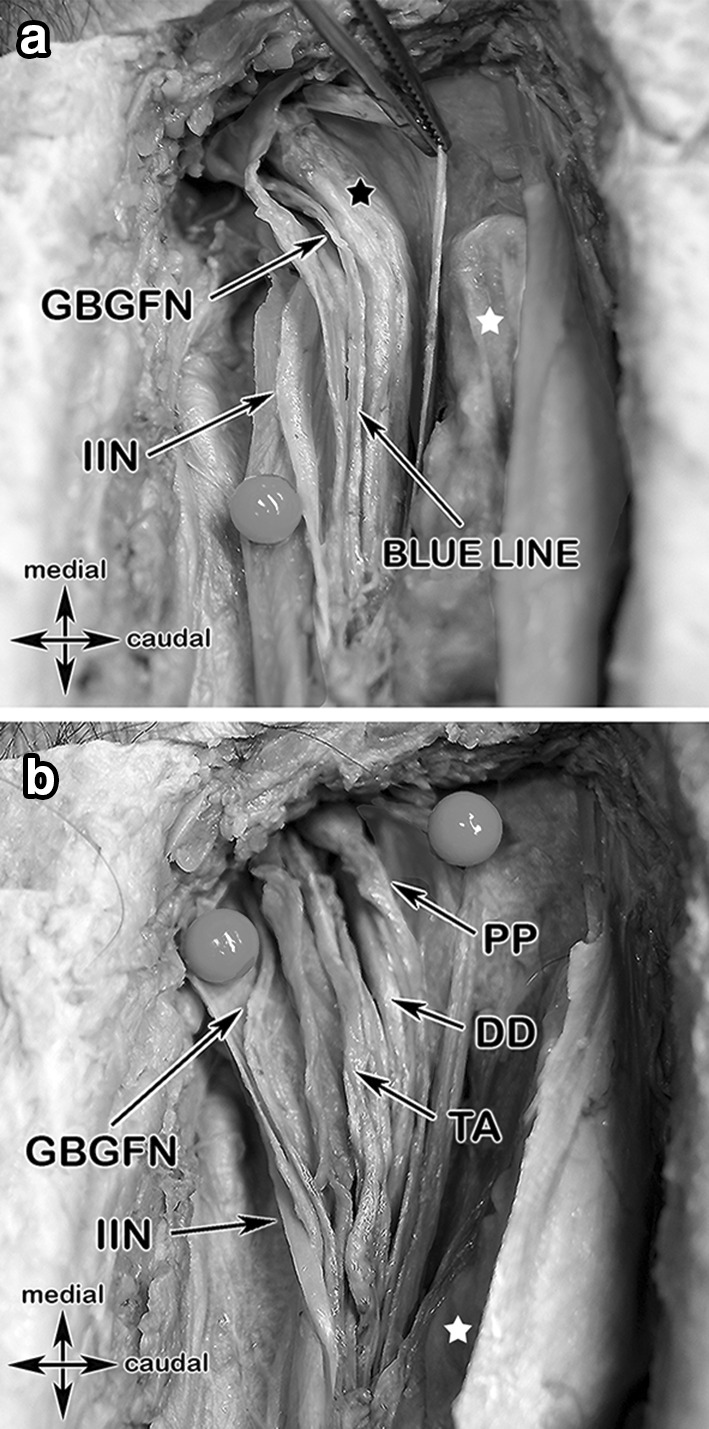
Colored anatomical specimen and a surgical case showing the spermatic cord layers and their topographic relationship to important structures in step 4. a The ilioinguinal nerve lying between the external spermatic fascia (purple) and the cremasteric fascia (green), the genital branch of the genitofemoral nerve is travelling along the posterior-medial aspect of the spermatic cord together with the cremasteric vein (“blue line”). b Opening the internal spermatic fascia (pink) exposes the deferens duct, pampiniform plexus and the testicular artery. c Surgical case with exposure of the ilioinguinal (lying dorsal of the spermatic cord), iliohypogastric and the genital branch of the genitofemoral nerves, each marked by a yellow vessel loop

In step 4 the spermatic cord was exposed and luxated. The IHN and the I-IN were marked with yellow vessel loops.

To find, identify and protect the GBGFN the layers of the spermatic cord were dissected:

- external spermatic fascia
  The I-IN lying superficial* to* the external spermatic fascia (the external spermatic fascia being the continuation of the External oblique aponeurosis)
- cremasteric fascia with cremasteric muscle fibers
- internal spermatic fascia

The GBGFN was identified within the cremasteric fascia and was accompanied by the cremasteric vein, denoted as “blue line”*.*

#### Step 5: Ductus deferens (DD), testicular artery (TA), pampiniform plexus (PP) (Fig. 8c)

In step number 5, furthermore the ductus deferens (DD), the testicular artery (TA) and the pampiniform plexus (PP) were identified within the internal spermatic fascia.

**Fig. 9:**
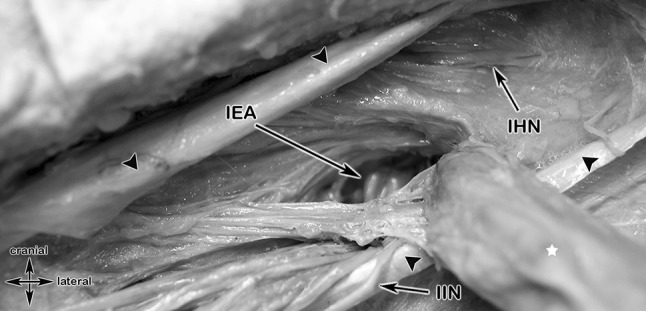
Anatomic specimen showing the topographic relationship of an indirect, lateral inguinal hernia (after splitting of the transversalis fascia) to the inferior epigastric artery, the iliohypogastric and ilio-inguinal nerve and the structures of the spermatic cord. Black arrow heads, External oblique aponeurosis; white star, indirect inguinal hernia; IEA, inferior epigastric artery; IHN, ilihypogastric nerve

The topographic relationship in the cadaver of the structures of the spermatic cord, the IHN and the I-IN is shown in Fig. 9.

### Immunohistochemistry

**Fig. 10:**
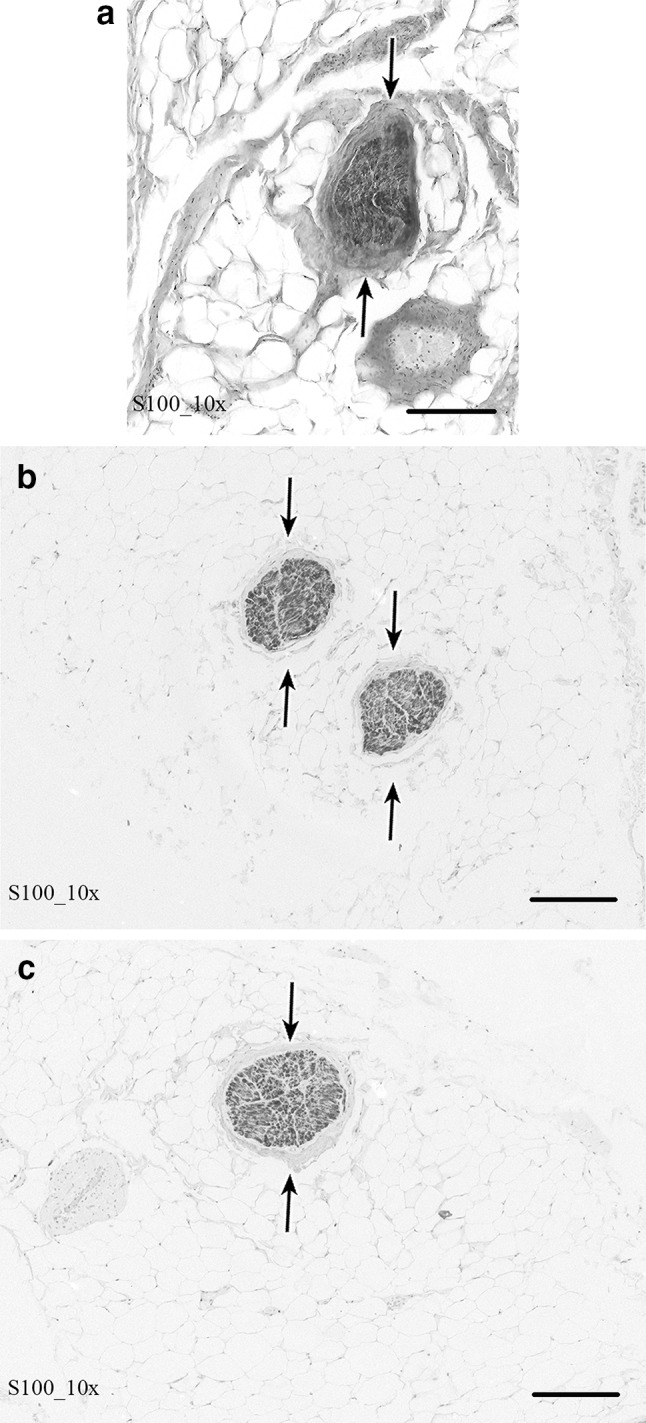
Microscopic section (200 μm) and immunohistochemistry using S100-antibody of the nerve branches of the GBGFN, IHN and I-IN of the respective inguinal area of the specimen

Figure 10a–c shows the results of the immunohistochemistry using S100-antibody of the presumed nerve tissue confirming nerve tissue (IHN, I-IN, GBGFN) in 100% of the cases.

### Ultrasound visualization: algorithm for practical usage (Figs. 1, 2, 11a, b)

**Fig. 11:**
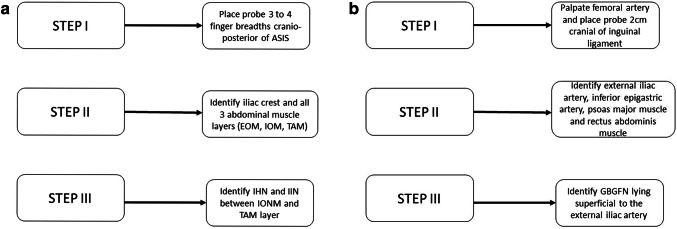
a Algorithm for preoperative ultrasonographic depiction of the IHN and I-IN. b Algorithm for preoperative ultrasonographic depiction of the GBGFN cranial to inguinal ligament

#### Iliohypogastric, Ilio-inguinal and Genital branch of the genitofemoral nerve

We developed an algorithm to depict all the relevant structures for preoperative or diagnostic ultrasonography (Fig. 11a, b).

## Discussion

In 2008, more than 20 million people globally were treated surgically for an inguinal hernia [38]. Already in 1993 the National Center for Health Statistics reported approximately 700,000 groin herniorrhaphies per year in the USA only, with an increasing incidence [38]. Data from the Netherlands (32,000 inguinal hernia repairs per year) and from Germany (275,000 inguinal hernia repairs per year) show that inguinal hernia surgery represents one of the most frequently performed interventions in general surgery [19]. Therefore, proficiency in neuroanatomy of the inguinal region and in surgical topography of the inguinal canal itself should be regarded as an indispensable basic requirement for all surgeons operating in this region.

The evidence shows that this region, from an anatomical–surgical point of few, seems to be a difficult and unfortunately poorly understood topographic region of the human body [18, 19, 39]. Additionally, only a few studies exist discussing pure “anatomic knowledge-gaps*”* and therefore could offer solutions to avoid nerve damage and chronic groin pain [18, 19]. The inguinal region provides a lot of potential surgical pitfalls, which can lead to appreciable impairments of patient´s everyday life. To avoid some of these anatomic pitfalls and to make open inguinal operations safer and more comprehensible this work offers a feasible step-by-step mapping of the surgical anatomy for daily routine application.

**Fig. 12:**
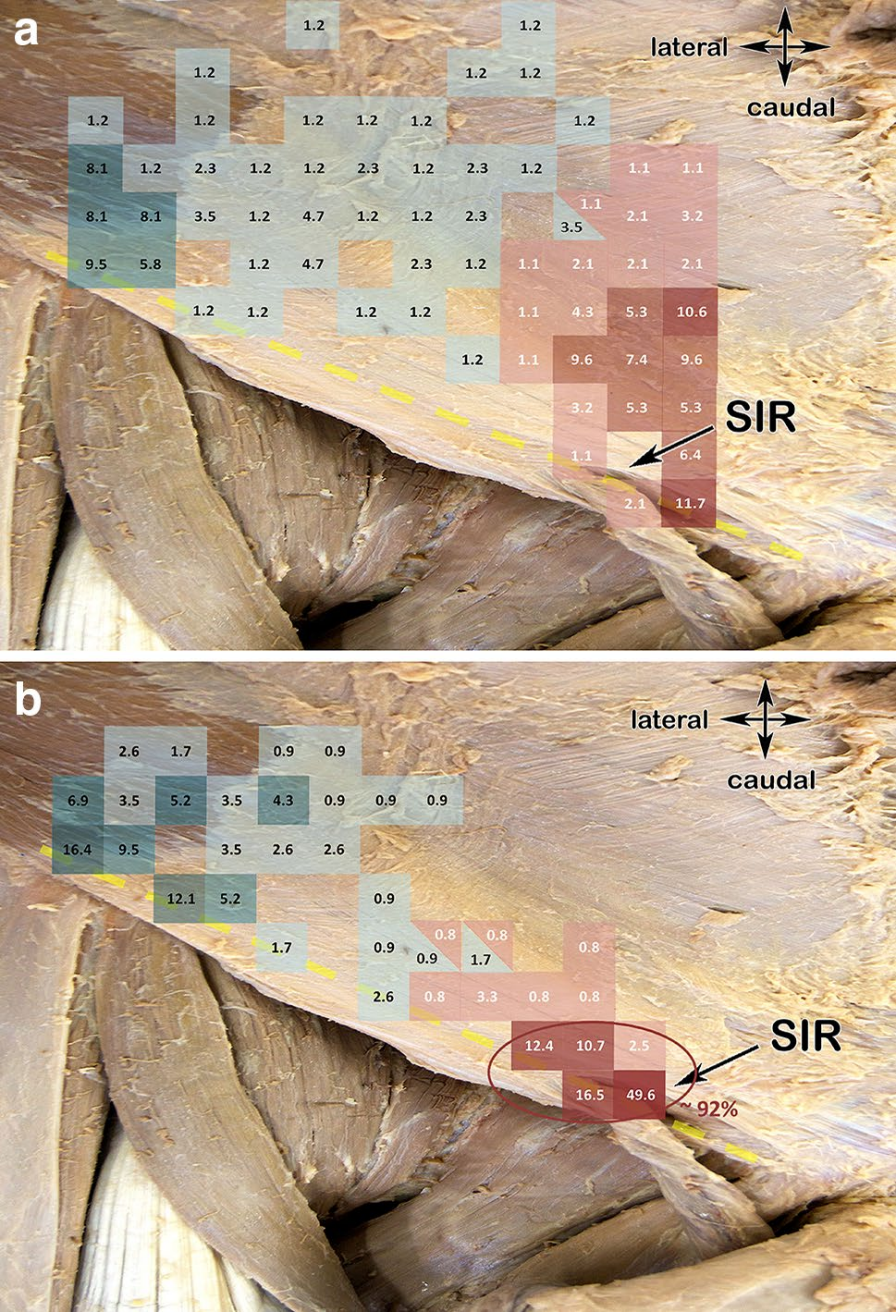
**a** Anatomical specimen including a colored scale showing the pattern representing the sensory IHN branches piercing the External oblique aponeurosis (based and adapted from Jamieson et al). Yellow dashed line, inguinal ligament; SIR, superficial inguinal ring. **b** Anatomical specimen including a colored scale showing the pattern representing the sensory I-IN branches piercing the External oblique aponeurosis (based and adapted from Jamieson et al). Yellow dashed line, inguinal ligament; SIR, superficial inguinal ring

Several studies, like the work of Hakeem et al., hypothesized that the main reasons for chronic groin pain are peri-operative nerve damage, post-operative fibrosis, or mesh-related fibrosis, classified as either neuropathic or nociceptive pain [40], involving all three inguinal nerves. Smeds et al. concluded that injury is due to inadequate dissection, failure to visualize and protection of the nerves, and failure to recognize the aberrant location and anatomic variations of the nerves [41]. The anatomical relationships of the different layers to the three primary inguinal nerves including ultrasonographic visualization could be shown in this study. Additionally, the medial, sensory cutaneous branches of IHN and I-IN, investigated by Jamieson et al., piercing the External oblique aponeurosis at variable topographic points are also important to bear in mind during the first steps of an open inguinal hernia repair [33, 42] (Fig. 12a, b); for the I-IN most constantly at the subcutaneous area of the superficial inguinal ring (around 92%) (Fig. 12a). The IHN’s perforation patterns through the aponeurosis of the external oblique are more varied but most are concentrated to an area above the medial one-third of the inguinal ligament (Fig. 12b).

Fränneby et al. predicted possible factors that predispose to post-operative inguinodynia: age below median, absence of a visible bulge before operation, recurrent hernia repair and history of moderate to severe pre-operative groin pain [43].

However, even though there are several studies in literature concerning different reasons of chronic groin pain [3, 5, 6, 13–17, 21, 44–49], no study exists, which had discussed and/or approved a pure lack of anatomic knowledge and/or understanding of the inguinal region by the respective surgeon. The studies of Lange et al. [18] and Wijsmuller et al. [19] address intraoperative nerve identification of all three inguinal nerves (IHN, I-IN, GBGFN) based on surgical-anatomic observations supporting the importance of appropriate anatomic knowledge. Although the recognition of all three inguinal nerves is strongly advocated [50] and evidence confirms that intraoperative identification of all three inguinal nerves decreases the risk of post-operative chronic groin pain (to less than 1%) [1, 18, 44, 50] only a minority of surgeons follow the recommendations of nerve-recognizing inguinal hernia surgery [1, 51]. The main reasons for doing so might be anatomic complexity and/or just a lack of topographic knowledge and therefore operation time is scarce [39].

Lange et al. [18] showed that identifying all three nerves, thorough anatomical knowledge and anatomical training assumed, will only add 3–4 min to the operating time for IHN and I-IN and 1–5 min for GBGFN. These 9 min extra time seem to be minimal, especially since the benefit for patients of identifying all three nerves is clear [18].

Although chronic groin pain is well established as a multifactorial process [1, 44], important basic requirements for an efficient, non-iatrogenic injury nerve-recognizing hernia repair include anatomical knowledge combined with anatomical training. Such training should be performed in specialized postgraduate clinical-anatomic training centers taught by clinically and surgically versed anatomists together with surgeons experienced in hernia repair. These postgraduate anatomic dissection courses, despite several critical annotations [52–54], using bequeathed bodies, are a highly valuable and indispensable component of (under- and) postgraduate medical education (and research) for all surgeons [29, 55] and might therefore be of possible value to prevent chronic groin pain caused by anatomic ignorance.

In contrast, Bischoff et al. also evaluated the risk of nerve damage and persistent pain for a nerve-identifying open herniorrhaphy, postulated no differences in regard to sensory loss (applying quantitative sensory testing, QST) and groin pain between patients groups with and without nerve identification during surgery 6 month after surgery [45]. However, the reported rate of nerve identification in this study was lower with around 80% of the GBGFN unidentified, and for the IHN and I-IN the proportions were 5.3% and 2.5%, respectively [45]. This low rate of identification of the GBGFN might confirm the argument of a lack of anatomical knowledge in this region combining with a lack of routine dissection in this area. They still found that around 16% of the patients with or without nerve-identification had a substantial pain-related impairment [45]. In the literature, other studies including Reinpold et al. in their long-term prospective cohort study recommend nerve visualization as a means to reduce chronic postherniorrhaphy pain [48] while also addressing that factors other than nerve identification may be of importance.

In our perspective, nerve visualization should also be done by performing an ultrasonographic visualization of all three inguinal nerves by surgeons themselves preoperatively, as shown in this study. Even if such an approach in practice might be hard to implement, especially for surgeons who are not proficient and experienced enough in ultrasonography, our “Points of optimal visibility” (POV’s) provided in this study, could be excellent landmarks for a safe and quick identification and delineation of the topographic course of these peripheral nerves in daily routine practice. This might lead to a less time-consuming nerve-identification surgery with the potential for better outcome regarding postoperative chronic groin [32]. Nevertheless, also in this specialization, postgraduate training in sonographic peripheral nerve topography might be of utmost importance; therefore, more large-scaled prospective studies would be required to determine which patients truly would benefit from such an approach.

## Conclusion

A detailed comprehension of inguinal anatomy and precise clinical–anatomical knowledge is an indispensable basic requirement for all surgeons. It is desirable to perform inguinal ultrasonography to identify the nerves or alternatively visually identify the nerves during an open inguinal hernia repair to avoid complications including postoperative inguinodynia and optimize patient outcomes.
